# Supplementary material for: Single-molecule imaging reveals distinct elongation and frameshifting dynamics between frames of expanded RNA repeats in C9ORF72-ALS/FTD
Source: Nat Commun. 2023 Sep 11;14:5581. doi: 10.1038/s41467-023-41339-x (PMC10495369; doi:10.1038/s41467-023-41339-x)
Supplement: Supplementary file 1 — Supplemetary Information [file 41467_2023_41339_MOESM1_ESM.pdf]

Single molecule imaging reveals distinct elongation and frameshifting dynamics between frames of expanded RNA repeats in C9ORF72-ALS/FTD

Authors: Malgorzata J. Latallo[1,2*], Shaopeng Wang[1,3,4*], Daoyuan Dong[3,4*], Blake Nelson[1,2], Nathan M. Livingston[1,2], Rong Wu[3,4], Ning Zhao[5], Timothy J. Stasevich[5], Michael C. Bassik[6], Shuying Sun[2,3,4,7#], Bin Wu[1,2,7#]

Supplementary Figures

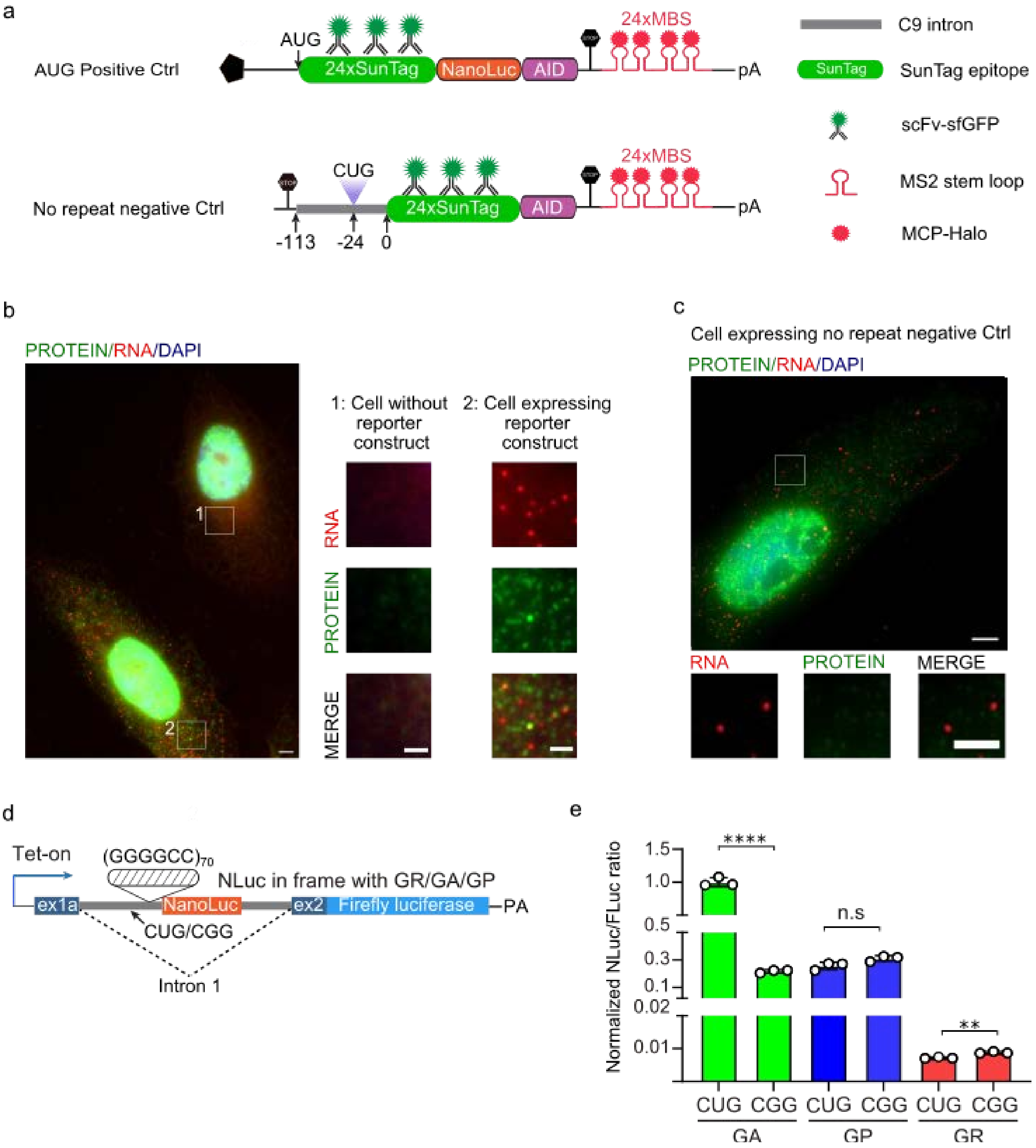

Supplementary Fig. 1 Single molecule imaging of RAN translation showed higher initiation rate of poly-GA than poly-GP and poly-GR

a. Schematic of constructs for single molecule imaging of RAN translation. The AUG positive control has a canonical AUG start codon in-frame with 24xSunTag, followed by Nano Luciferase and AID. The no repeat (negative) control contains 113-nucleotide endogenous intron sequence upstream of the GGGGCC repeats. A CUG near-cognate start codon (-24 bp) is in frame with GA. There are multiple stop codons in the intron sequence that prevent leaky cap-dependent translation. **b.** Representative smFISH-IF image of control cells with and without reporters. Cell 1 did not express the reporter and had no visible RNA signals (red puncta) or single proteins (green puncta). In comparison, cell 2 in the same image expressed reporter and had both RNA and protein signals. Scale bar: 5μm, zoom in 2.5μm. Numbers of quantified cells are listed in Fig. 1c. **c.** Representative smFISH-IF image of no-repeat negative control cell. Although there were RNA signals (red puncta) , there were rarely protein signals (both mature single proteins and translation sites). Red: RNA; Green: protein. Scale bar: 5μm, zoom in 2.5μm. Numbers of quantified cells are listed in Fig. 1c. **d.** Schematic of the minigene for C9ORF72 intron 1 with luciferase to report the RAN translation product. The NanoLuc was inserted after GGGGCC repeat with zero, one or two nucleotides offset to measure the RAN translation of GA/GP/GR respectively. In addition, the CUG near-cognate start codon 24-nucleotide upstream of the repeat was mutated into CGG for each frame separately. All reporters were stably integrated into HeLa Flp-In cells separately. **e.** Relative RAN translation products were compared after 24 h induction. NLuc signals were divided by FLuc signal in each sample to normalize the expression level. To compare the relative expression in each frame, all DPR-NLuc signals are normalized to the CUG-GA frame. Data are mean ± SD from three biological replicates. Two-tailed Student t-test was performed to compare the CUG-DPR vs CGG-DPR in each group. GA, ****P=0.0004; GP, P=0.051; GR, **P=0.0018.

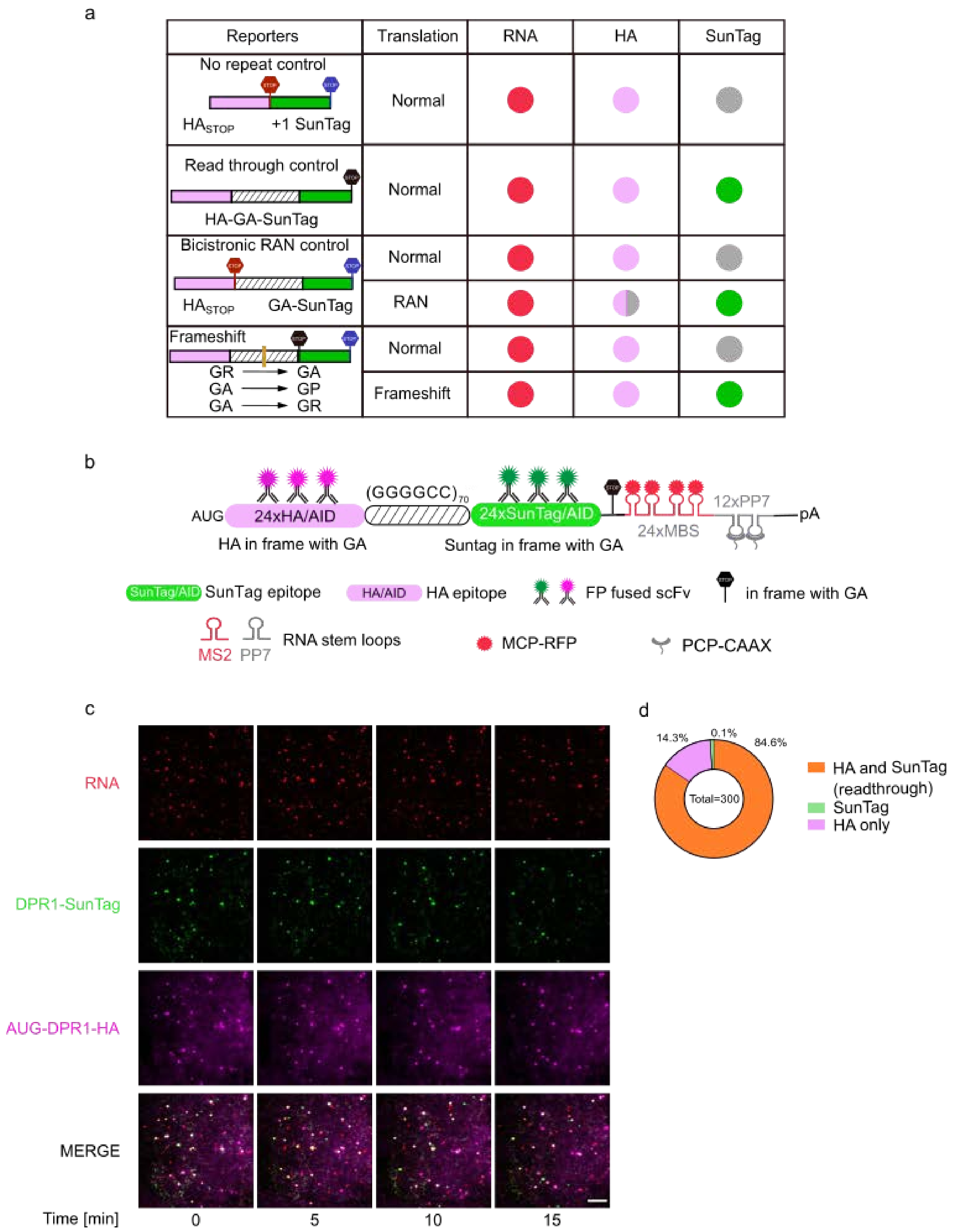
a
Reporters
Translation
RNA
HA
SunTag
No repeat control
HASTOP
+1 SunTag
Normal
Read through control
HA-GA-SunTag
Normal
Bicistronic RAN control
HASTOP
GA-SunTag
Normal
RAN
Frameshift
GR → GA
GA → GP
GA → GR
Normal
Frameshift
b
AUG
24xHA/AID
(GGGGCC)70
24xSunTag/AID
12xPP7
pA
24xMBS
HA in frame with GA
Suntag in frame with GA
SunTag/AID SunTag epitope
HA/AID HA epitope
FP fused scFv
in frame with GA
MS2 PP7 RNA stem loops
MCP-RFP
PCP-CAAX
c
RNA
DPR1-SunTag
AUG-DPR1-HA
MERGE
Time [min]
0
5
10
15
d
14.3%
0.1%
84.6%
Total=300
HA and SunTag (readthrough)
SunTag
HA only

Supplementary Fig. 2 The two-color translation assay efficiently reports translation readthrough.

a. All reporters and controls used to study frameshift. The expected signals in each fluorescence channel are pseudo-color coded. Red: RNA; Magenta: HA; Green: SunTag; Grey: No signal in the channel. The frameshift reporters are described in Fig. 2. The no-repeat control has a stop codon in the HA frame. The bicistronic RAN translation control reporter has stop codons in each reading frame before the repeats (Fig. S3). **b.** Schematic of the readthrough control construct: SunTag was placed in frame with AUG-HA and poly-GA with no stop codon in this frame. **c.** Time-lapse images illustrating translation dynamics of the readthrough control reporter. Red (RNA): stdMCP-RFP; magenta (protein): HA-Fb-HaloTag; green (Protein): SunTag-scFv-sfGFP. About 84.6% of translating molecules have visible signals in both HA and SunTag channels, illustrating capability and precision of the single-molecule two-color assays. Also see Supplemental Movie S4. **d.** Quantification of readthrough control translation. The number of translation events with SunTag and HA signals was divided by the total number of translating events coming from all RNAs.

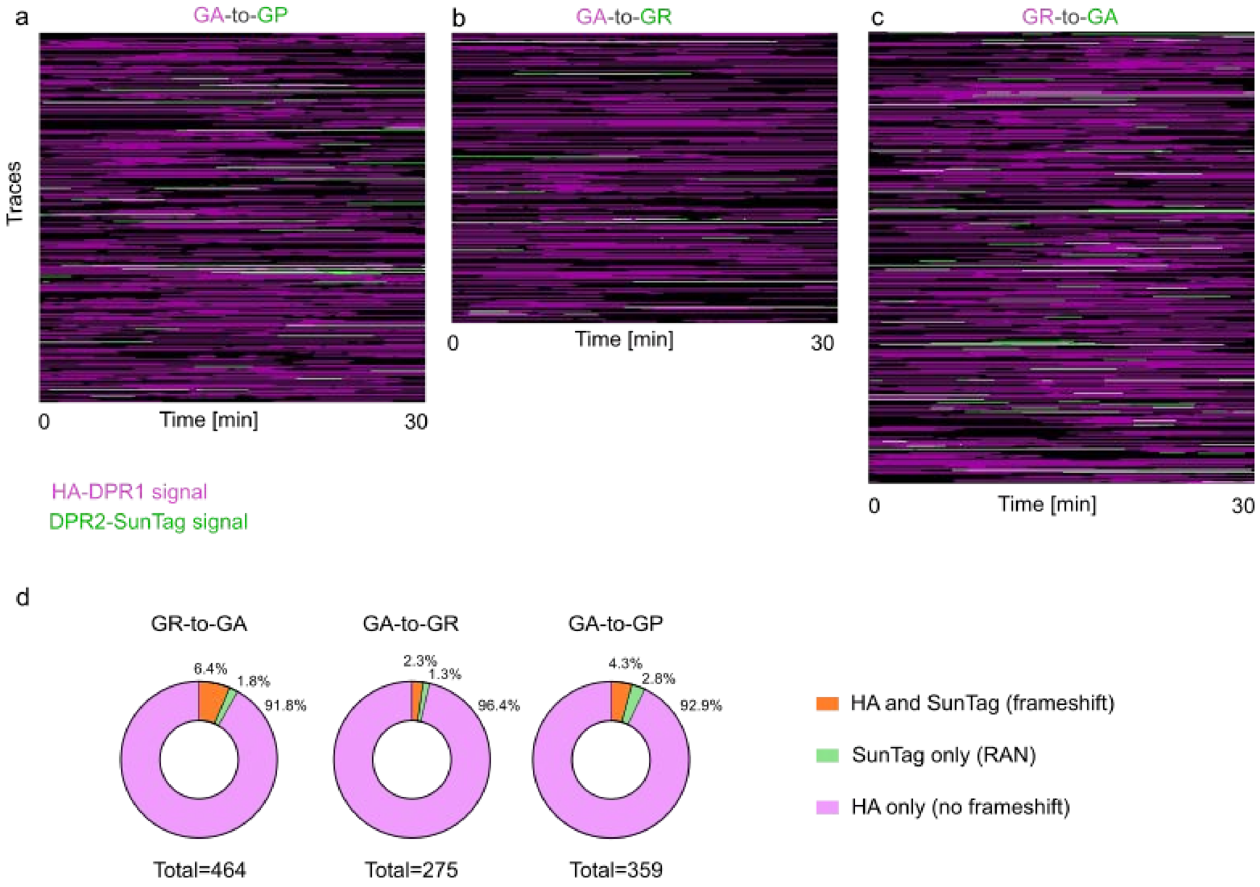

Supplementary Fig. 3 Combined fluorescence traces of translated mRNAs for all frameshifting reporters.

a. GA-to-GP reporter. **b.** GA-to-GR reporter. **c.** GR-to-GA reporter. Magenta (protein): HA-Fb-HaloTag indicating the translation of HA-DPR1; green (Protein): SunTag-scFv-sfGFP indicating the translation of DPR2-SunTag after frameshifting. **d.** Percentage of time of mRNAs undergoing normal translation (only in HA-DPR1 frame, magenta), frameshift (both frames, orange), or RAN translation (only DPR2-SunTag frame, green). The time during which mRNA was in a specific state was divided by the total translation time for the track to calculate the percentage: GR-to-GA (5 cells, 464 translation events); GA-to-GR (6 cells, 275 translation events); GA-to-GP (6 cells, 359 translation events).

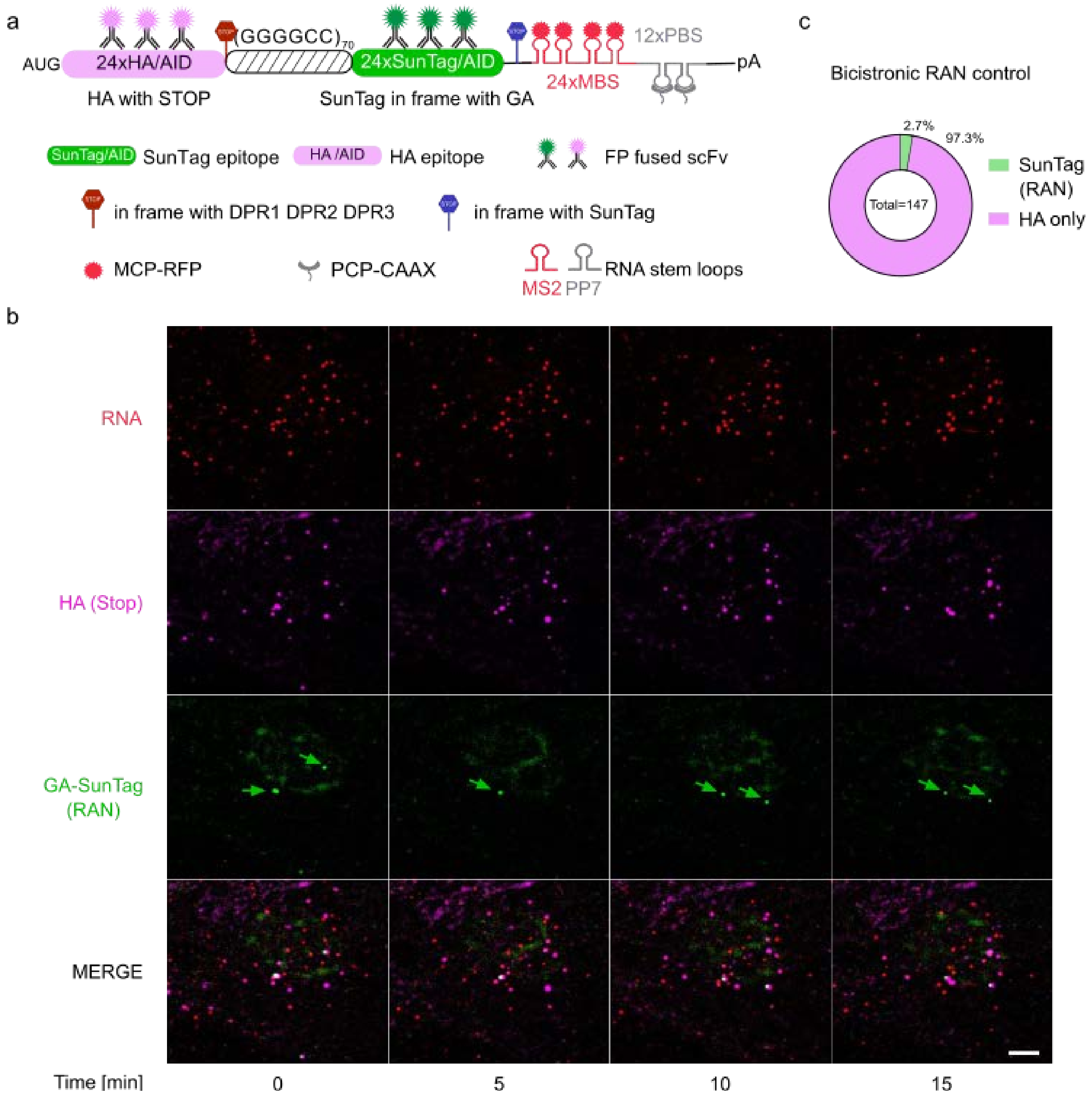

Supplementary Fig. 4 RAN translation events are rare in the two-color single molecule bicistronic RAN translation control reporter.

a. Schematic of the bicistronic RAN translation control construct. **b.** Time-lapse images illustrating translation dynamics of RAN translation control reporter. Red (RNA): stdMCP-RFP; magenta (protein): HA-Fb-HaloTag; green (Protein): SunTag-scFv-sfGFP. See Supplemental Movie S6 also. **c.** Quantification of RAN translation frequency in the bicistronic RAN control reporter. The number of translation events with only SunTag signal was divided by the total number of translation events from all RNAs.

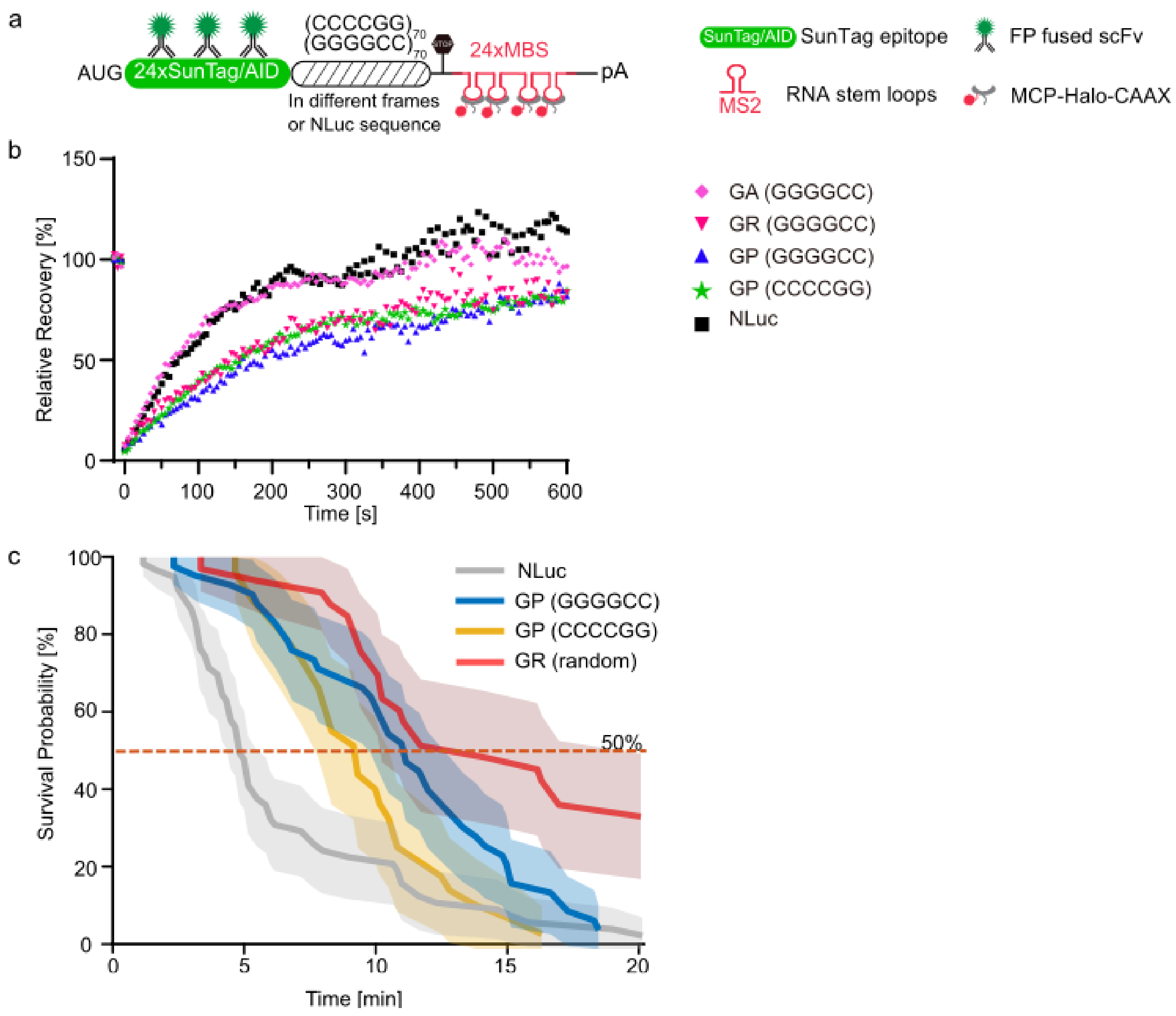

Supplementary Fig. 5 Amino acid sequence of the DPRs influences the elongation speed. **a**. Diagram of the single-molecule DPR elongation reporter construct. The $(GGGGCC)_{70}$ or $(CCCCGG)_{70}$ fragment was inserted directly after SunTag in different reading frames. **b**. The normalized fluorescence intensities for single molecule fluorescence recovery after photobleaching (FRAP) experiment for indicated reporters. Single translation sites were bleached with a focused laser, then tracked for 10 minutes to measure recovery. The fluorescence recovery was normalized to the translation site intensity before bleaching. The numbers of translation sites analyzed for each condition are: NLuc: 36; GA(GGGGCC): 36; GP(GGGGCC): 23; GP(CCCCGG): 35; GR(GGGGCC): 26. **c**. The survival curves for ribosome run-off experiments for NLuc, poly-GR random and poly-GP encoded by different sequences. The dashed line represents the 50% of mRNAs that have finished runoff. The shadowed area represents the 95%

confidence bounds (Greenwood's formula). The numbers of translation sites (in 4-6 cells) analyzed were: GR(random): 33; GP(GGGGCC): 42; GP(CCCCGG): 27; NLuc: 60.

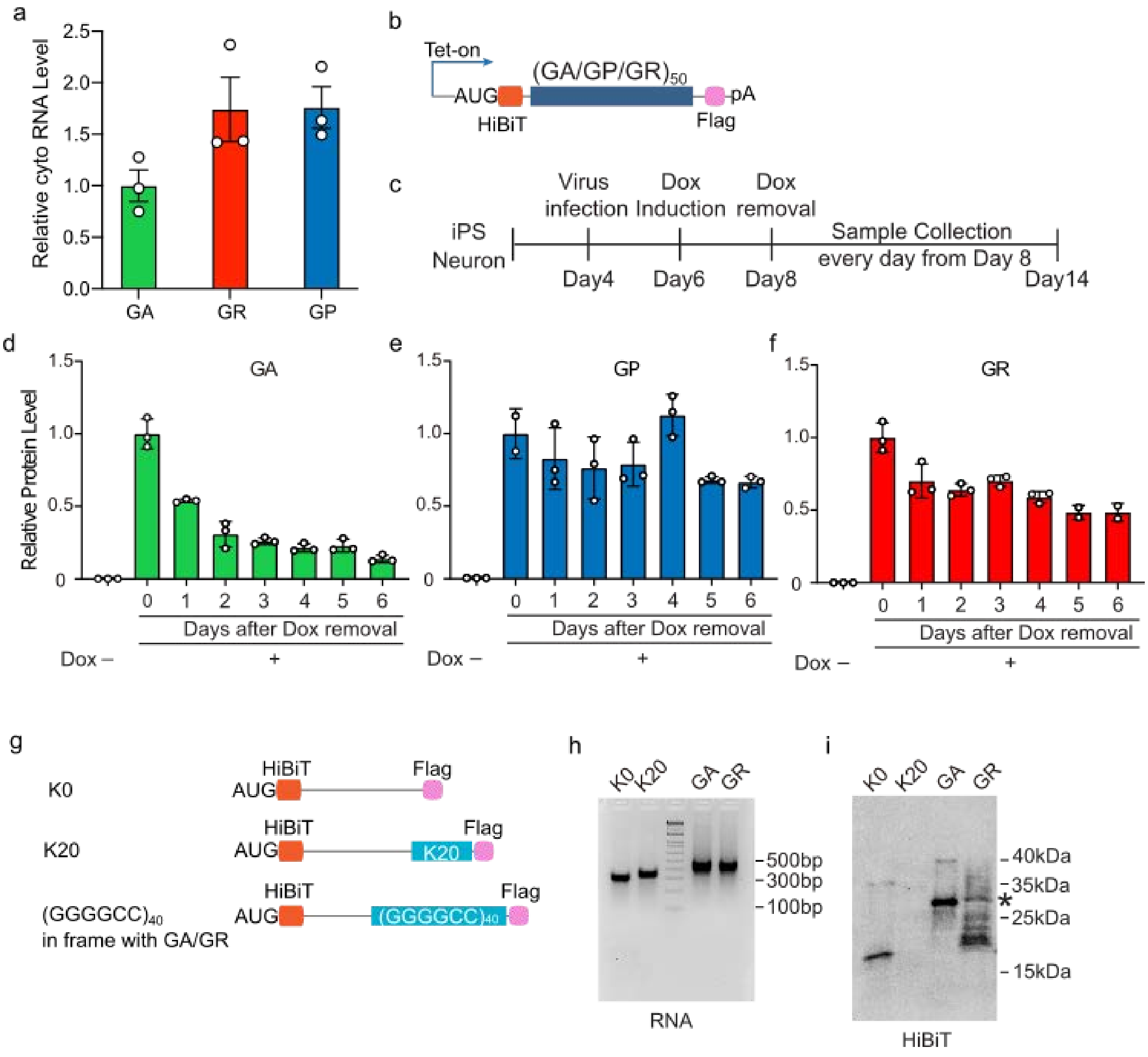

Supplementary Fig. 6 The translation elongation of poly-GR is stalled.

a. Reporter RNA levels for the experiment in Fig. 4 were measured by qRT-PCR. Data were reported as mean ± SEM from three biological replicates. **b-f.** DPR stability is not the major driver for the difference in DPR level. **b.** The lentiviral construct for inducible expression of HiBiT-tagged DPRs. **c.** Workflow for measuring the stability of different DPRs in iPS-neurons. **d-f**. The relative level of DPR proteins in iPS-neurons at different time points after stopping induction. The relative protein level was calculated by normalizing the activity of HiBiT to the total protein level measured by BCA assay, and the value at each time point was normalized to Day 0 after Dox removal. Data reported mean ± SD from two or three biological replicates. **g-i**. *In vitro* translation of different elongation reporters. **g.** Diagram of *in vitro* translation reporters. The K0 reporter has

no stalling sequence between HiBiT and Flag. The K20 (20× AAA which encodes 20× lysine) and $GGGGCC_{40}$ encoding GA or GR were in frame with AUG-HiBiT and Flag tag. **h.** Representative agarose gel showed the uniform size and high purity of *in vitro* transcribed RNA used for the translation assay. Equal amounts of RNA were used for each reaction. Three independent experiments were done with similar results obtained. **i.** Representative HiBiT blot of translation products in rabbit reticulocyte lysate (RRL). There was no detectable protein in the K20 construct because of the strong RQC effect for poly-A sequence. The GR-$GGGGCC_{40}$ reporter showed extensive truncated products. * indicates the full-length proteins. Three independent experiments were done with similar results obtained.

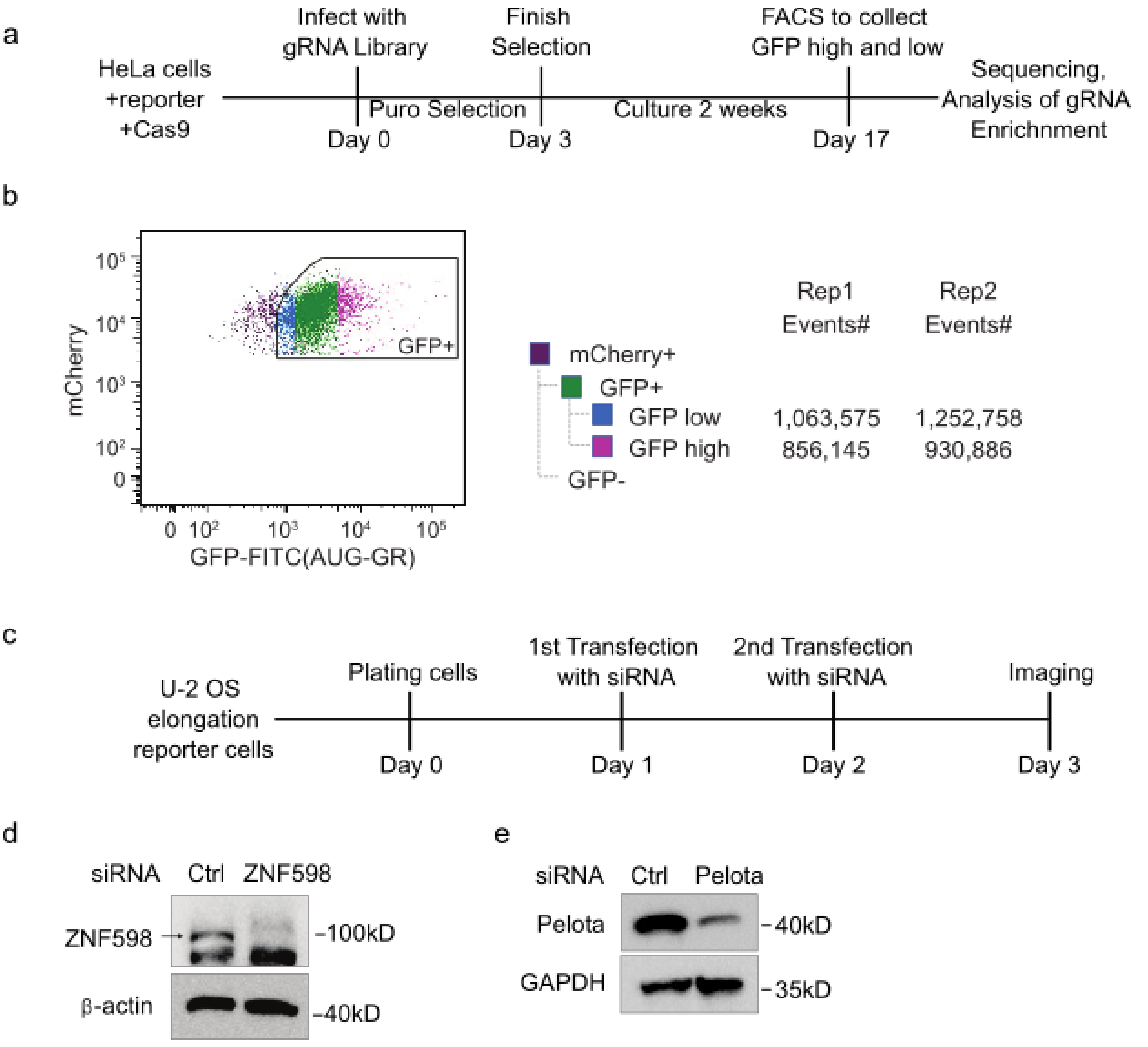

Supplementary Fig. 7 The RQC factors modulate translation elongation dynamics through repeat RNAs.

a. Workflow of CRISPR-Cas9 knockout screen for factors influencing GR-GFP level (Fig. 6a-b). Hela Flp-In cells with stably integrated GR-GFP reporter were infected with lentiviral gRNA library. The GFP-high and GFP-low cell populations were collected by FACS, and subjected to deep sequencing. Statistical analysis was performed to identify gRNAs enriched in each population. The result is shown in Fig. 6b. **b**. Gating strategy for FACS. Cells of interest were first gated by FSC and SSC, and mCherry+ cells (purple) were selected for sgRNA expression. From GFP+ cells (green), the top 5% GFP-low (blue) and top 5% GFP-high (pink) populations were collected, which correspond to the GR-GFP low and GR-GFP high groups in Figure 6b, respectively. The

number of events collected was indicated in the figure. **c.** Timeline for siRNA knockdown and ribosome runoff experiment. After 2 days of siRNA treatment, ribosome run-off experiments were performed. **d-e**. Representative western blot demonstrates the knockdown of ZNF598 (d) and Pelota (e) in U-2 OS cells using siRNA. Two independent experiments were done with similar results.
