# Supplementary material for: Single-molecule imaging reveals distinct elongation and frameshifting dynamics between frames of expanded RNA repeats in C9ORF72-ALS/FTD
Source: Nat Commun. 2023 Sep 11;14:5581. doi: 10.1038/s41467-023-41339-x (PMC10495369; doi:10.1038/s41467-023-41339-x)
Supplement: Supplementary file 3 — Description of Additional Supplementary Files [file 41467_2023_41339_MOESM3_ESM.pdf]

Description of Additional Supplementary Files

File Name: Supplementary Movie 1
Description: **A representative movie showing RAN translation in the GA frame (related to Fig.1).**
U-2 OS cells were transfected with GA RAN translation reporter (Fig. 1a). Green: translation sites (SunTag signal); red: RNA. For long-term imaging, RNA was tethered to the cell membrane (see Methods). A selected portion of the cell shows translation sites colocalized with mRNA. Images were taken every 5s for the green channel and every 15s for the red channel for 30min, and movie represents a 5 min snapshot. Scale bar: 5μm.

File Name: Supplementary Movie 2
Description: **A representative movie comparing the translation of wild type CUG-GA and the mutant CGG-GA RAN translation.**
U-2 OS cells were transiently transfected with GA or GA CGG reporters (Figs. 1a, g-f). Green: translation sites (SunTag signal); red: RNA, RNA was tethered to the cell membrane for long-term imaging. The fraction of translation time for CUG-GA reporters is longer than that of CGG-GA. Images were taken every 5s for the green channel and every 15s for the red channel for 30min, and movie represents a 5 min snapshot. Scale bar: 5μm.

File Name: Supplementary Movie 3
Description: **A representative movie for the frameshift negative control reporter.**
There is no GGGGCC repeats between HA-tag and SunTag, and 3 stop codons were placed after HA-tag, one in each frame. Left: merge of all channels. Right: green: +1 SunTag frame translation (from frameshift); magenta: AUG-HA-tag translation; red: RNA. Frameshift events were not observed in the movie: no SunTag signal colocalized with RNA and HA-tag. Images were taken every 5s for the green and magenta channels and every 15s for the red channel for 30min, movie represents a 15min snapshot. Scale bar: 5μm.

File Name: Supplementary Movie 4
Description: **A representative movie showing the readthrough reporter (Fig. S2b-c).**
The HA-tag, $(GGGGCC)_{70}$ in the GA frame and SunTag were fused together in one reading frame without any stop codon. Majority of translating mRNAs colocalized with both SunTag and HA-tag signal. Green: SunTag frame translation (in frame with GA); magenta: AUG-HA-tag (in frame with GA), red: RNA, RNA was tethered to the cell membrane for long-term imaging. Images were taken every 5s for the green and magenta channels and every 15s for the red channel for 30min, movie represents a 5 min snapshot. Scale bar: 5μm. Scale bar: 5μm.

File Name: Supplementary Movie 5
Description: **A representative movie showing repeats-induced frameshifting.**
The GR-to-GA frameshift reporter (Fig. 2a) was stably expressed in U-2 OS cells. Green: GA DPR translation (SunTag); magenta: AUG-HA translation in GR frame; red: RNA, RNA was tethered to the cell membrane for long-term imaging. Most mRNAs colocalize with AUG-driven HA-tag. Transient SunTag signal colocalized with both HA tag and mRNA, indicating a frameshift event. Images were taken every 5s for the green and magenta channels and every 15s for the red channel for 30min, movie represents a 10 min snapshot Scale bar: 5µm. Scale bar: 5µm.

File Name: Supplementary Movie 6
Description: **A representative movie for the dual-color bicistronic RAN translation control reporter cells (Fig S4).**
U-2 OS cells stably expressing RAN translation control construct, with 3 stop codons placed after HA-tag, one in each frame preventing ribosomal readthrough, therefore any green fluorescence signal from +1 frame are indication of RAN translation initiation. A merge of all channels of selected portion of the cell shows translation sites colocalized with mRNA and single channels. Green: translation sites, red: RNA, RNA was tethered to cell membrane for long term imaging (see Methods). Images were taken every 5s for the green channel and every 15s for the red channel for 30min, movie represents a 15 min snapshot. Scale bar: 5µm.

File Name: Supplementary Movie 7
Description: **Ribosome run off experiment of elongation reporter for all three reading frames.**
U-2 OS cells stably expressing GA or GP, or GR elongation reporters (Fig. 4a) were treated with Harringtonine and imaged one minute after adding the drug. Images were taken every 10s for 20min. A selected portion of cells from three different reporter cell lines showed different time of runoffs. Green: translation sites (SunTag signal); red: RNA, RNA was tethered to cell membrane for long term imaging. Scale bar: 5µm.

File Name: Supplementary Movie 8
Description: **Fluorescence recovery after photobleaching (FRAP) experiment for single translation sites.**
U-2 OS cells stably expressing different elongation reporters (Fig. 4a) were imaged to identify a translation site, which was then bleached with a focused 488nm laser. Images were taken after bleaching every 5s for protein and every 15s for RNA channel for total of 10 minutes. Red: RNA, tethered to the cell membrane; green: translation sites (SunTag signal). From the movie, GA translation sites recover faster after bleaching than GR translation sites.

File Name: Supplementary Movie 9
Description: **Ribosome run off experiment of elongation reporter after ZNF598 knockdown.** U-2 OS cells stably expressing GR elongation reporters (Fig. 4a) were treated with siRNAs targeting ZNF598 or scrambled control. The cells were treated with Harringtonine and imaged every 10s from 1min to 20min after adding the drug. Reducing ZNF598 prolonged the run-off time of GR translation sites. Green: translation sites (SunTag signal); red: RNA, tethered to the cell membrane. Scale bar: 5µm.

File Name: Supplementary Data 1
Description: CRISPR/Cas9 screen result for genetic modifiers of poly-GR production using the RBP sub-library.

File Name: Supplementary Data 2
Description: Patient-derived iPSC lines used for proteomic analysis, related to Figure 6
