# Supplementary material for: Single-molecule imaging reveals distinct elongation and frameshifting dynamics between frames of expanded RNA repeats in C9ORF72-ALS/FTD
Source: Nat Commun. 2023 Sep 11;14:5581. doi: 10.1038/s41467-023-41339-x (PMC10495369; doi:10.1038/s41467-023-41339-x)
Supplement: Supplementary file 15 — Reporting Summary [file 41467_2023_41339_MOESM15_ESM.pdf]

nature portfolio

Corresponding author(s): Bin Wu, Shuying Sun

Last updated by author(s): Jul 29, 2023

Reporting Summary

Nature Portfolio wishes to improve the reproducibility of the work that we publish. This form provides structure for consistency and transparency in reporting. For further information on Nature Portfolio policies, see our Editorial Policies and the Editorial Policy Checklist.

Statistics

For all statistical analyses, confirm that the following items are present in the figure legend, table legend, main text, or Methods section.

n/a	Confirmed	
☐	☒	The exact sample size (*n*) for each experimental group/condition, given as a discrete number and unit of measurement
☐	☒	A statement on whether measurements were taken from distinct samples or whether the same sample was measured repeatedly
☐	☒	The statistical test(s) used AND whether they are one- or two-sided<br>*Only common tests should be described solely by name; describe more complex techniques in the Methods section.*
☒	☐	A description of all covariates tested
☒	☐	A description of any assumptions or corrections, such as tests of normality and adjustment for multiple comparisons
☐	☒	A full description of the statistical parameters including central tendency (e.g. means) or other basic estimates (e.g. regression coefficient) AND variation (e.g. standard deviation) or associated estimates of uncertainty (e.g. confidence intervals)
☐	☒	For null hypothesis testing, the test statistic (e.g. *F*, *t*, *r*) with confidence intervals, effect sizes, degrees of freedom and *P* value noted<br>*Give P values as exact values whenever suitable.*
☒	☐	For Bayesian analysis, information on the choice of priors and Markov chain Monte Carlo settings
☒	☐	For hierarchical and complex designs, identification of the appropriate level for tests and full reporting of outcomes
☒	☐	Estimates of effect sizes (e.g. Cohen's *d*, Pearson's *r*), indicating how they were calculated

Our web collection on statistics for biologists contains articles on many of the points above.

Software and code

Policy information about availability of computer code

Data collection	Data imaging was performed using Nikon Elements v4.8. qPCR data were acquired using Bio-Rad CFX Manager v3.1. Western blotting data was acquired by BIO-RAD ChemiDoc imaging system. Sequencing was performed using Illumina NextSeq 500 High 150.
Data analysis	ingle-molecule FISH data were anylized using Matlab R2017a and FISH-Quanta v3. Analysis of live cell imaging was done using Airlocalize and u-track v2, visualization of the images was done using Fiji v1.8, Graph Pad Prism9.4. was used for plotting and data visualization. Sequencing data was analyzed using the pipeline https://bitbucket.org/dmorgens/castle.

For manuscripts utilizing custom algorithms or software that are central to the research but not yet described in published literature, software must be made available to editors and reviewers. We strongly encourage code deposition in a community repository (e.g. GitHub). See the Nature Portfolio guidelines for submitting code & software for further information.

Data

Policy information about availability of data

All manuscripts must include a data availability statement. This statement should provide the following information, where applicable:

- Accession codes, unique identifiers, or web links for publicly available datasets
- A description of any restrictions on data availability
- For clinical datasets or third party data, please ensure that the statement adheres to our policy

All sequencing data are available with the following link: https://www.ncbi.nlm.nih.gov/sra/PRJNA905075

Research involving human participants, their data, or biological material

Policy information about studies with human participants or human data. See also policy information about sex, gender (identity/presentation), and sexual orientation and race, ethnicity and racism.

Reporting on sex and gender	N/A
Reporting on race, ethnicity, or other socially relevant groupings	N/A
Population characteristics	N/A
Recruitment	N/A
Ethics oversight	N/A

Note that full information on the approval of the study protocol must also be provided in the manuscript.

Field-specific reporting

Please select the one below that is the best fit for your research. If you are not sure, read the appropriate sections before making your selection.

☒ Life sciences ☐ Behavioural & social sciences ☐ Ecological, evolutionary & environmental sciences

For a reference copy of the document with all sections, see nature.com/documents/nr-reporting-summary-flat.pdf

Life sciences study design

All studies must disclose on these points even when the disclosure is negative.

Sample size	No sample size calculation was performed for this work. All sample sizes were determined based on standard protocols in the field. Unless noted otherwise n=3 biological replicates was used.
Data exclusions	An earlier submitted two sets of data were replaced with newly collected data, current figure 1f). We ran an outlier test on all four GA biological replicates (previously collected three and one new). One of the old biological replicate sets was identified as an outlier (Grubb's test). We decided to remove that set and the corresponding GA CGG set, as that replicate is not representative due to the low number of data points. The current set comparison shows ** significance (P = 0.0071).
Replication	All experiments were repeated at least three times. The specific number of replicates is listed in each figure legends. All replication attempts were successful.
Randomization	Cells were plated randomly to a multi-well dish, they were assigned randomly to different experimental group. The images were taken at random positions.
Blinding	The data collection was not performed blindly, but the experimental results were repeated by different members of the research team.

Reporting for specific materials, systems and methods

We require information from authors about some types of materials, experimental systems and methods used in many studies. Here, indicate whether each material, system or method listed is relevant to your study. If you are not sure if a list item applies to your research, read the appropriate section before selecting a response.

Materials & experimental systems

n/a	Involved in the study
☐	☒ Antibodies
☐	☒ Eukaryotic cell lines
☒	☐ Palaeontology and archaeology
☒	☐ Animals and other organisms
☒	☐ Clinical data
☒	☐ Dual use research of concern
☒	☐ Plants

Methods

n/a	Involved in the study
☒	☐ ChIP-seq
☐	☒ Flow cytometry
☒	☐ MRI-based neuroimaging

Antibodies

Antibodies used: GFP (1:5000, Aves Labs, GFP-1010), GAPDH (1:1000, Cell Signaling Technology,, #2118), FLAG (1:1000, Sigma, #F1804), ZNF598 (1:1000, GeneTex, #GTX119246), PELO (1:1000, proteintech, #10582-1-AP), b-actin (1:1000, Cell Signaling, #3700)

Validation: All antibodies used in this work are commercially available and the validation can be accessed at manufactures website.

Eukaryotic cell lines

Policy information about cell lines and Sex and Gender in Research

Cell line source(s): U-2 OS (American Type Culture Collection HTB-96); HEK293T (American Type Culture Collection CRL-1573); HeLa (American Type Culture Collection CCL-2); HeLa Flp-In (Thermo Fisher); SH-SY5Y (American Type Culture Collection CRL-2266)

Authentication: All are from commercial resources and were previously published.

Mycoplasma contamination: Cell lines are tested monthly for mycoplasma using

Commonly misidentified lines (See ICLAC register): N/A

Flow Cytometry

Plots

Confirm that:

☐ The axis labels state the marker and fluorochrome used (e.g. CD4-FITC).

☐ The axis scales are clearly visible. Include numbers along axes only for bottom left plot of group (a 'group' is an analysis of identical markers).

☐ All plots are contour plots with outliers or pseudocolor plots.

☐ A numerical value for number of cells or percentage (with statistics) is provided.

Methodology

Sample preparation: Cells were dissociated and resuspended in sorting buffer (1xPBS, 25mM HEPES pH7.0, 1% FBS, Antibiotics) and analyzed by FACS

Instrument: Aria III Cell Sorter (BD Biosciences)

Software: FACS Diva (version 6.1.3, BD Biosciences) and FlowJo (version 10.0.7, FlowJo LLC)

Cell population abundance: More than 20 million cells were analyzed by FACS, sgRNA expressing cells was determined by mCherry PE-Texas Red signals, and reporter expression is determined by GFP-FITC signals. Around 1 million were collected for 10% GFP-high and GFP-low population individually from each replicate.

Gating strategy: The live cells were first gated using FSC-A/SSC-A. The singlets were then gated using FSC-H/FSC-W and SSC-H/SSC-W. sgRNA expressing cells were gated using mCherry PE-Texas Red. GR-GFP expression is gated by GFP-FITC. Top 10% GFP-high and 10% GFP-low cell populations were collected. The cells without reporter or Cas9-BFP expression were used as negative control.

☐ Tick this box to confirm that a figure exemplifying the gating strategy is provided in the Supplementary Information.
